# Supplementary material for: SARS-COV-2 antibody responses to AZD1222 vaccination in West Africa
Source: Nat Commun. 2022 Oct 17;13:6131. doi: 10.1038/s41467-022-33792-x (PMC9574797; doi:10.1038/s41467-022-33792-x)
Supplement: Supplementary file 1 — Supplementary file [file 41467_2022_33792_MOESM1_ESM.pdf]

a

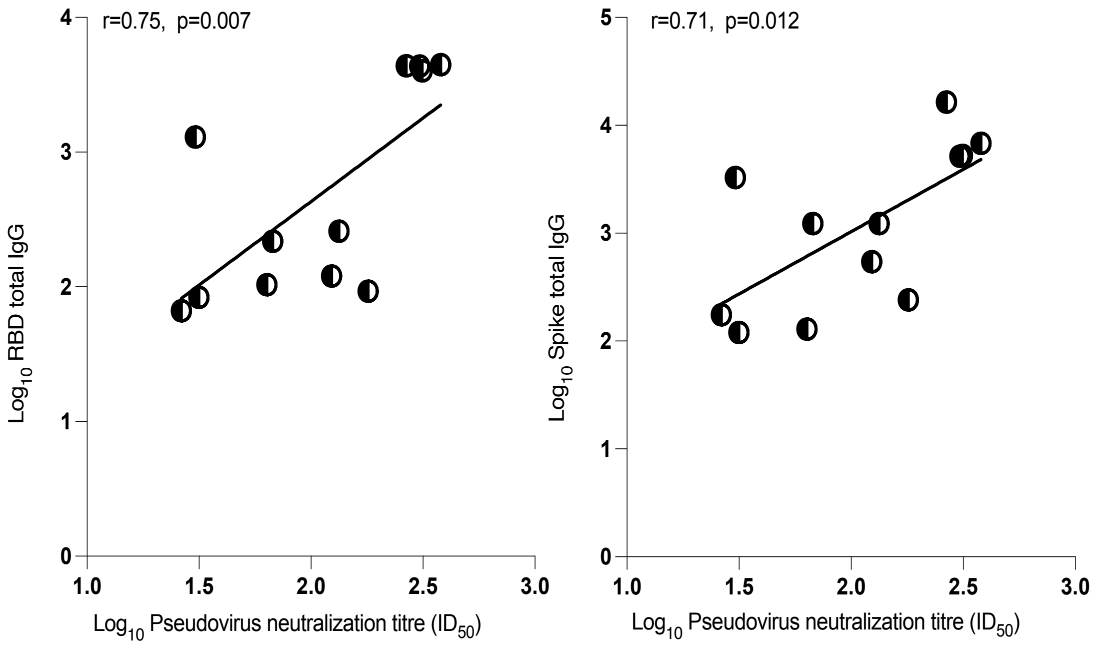

b

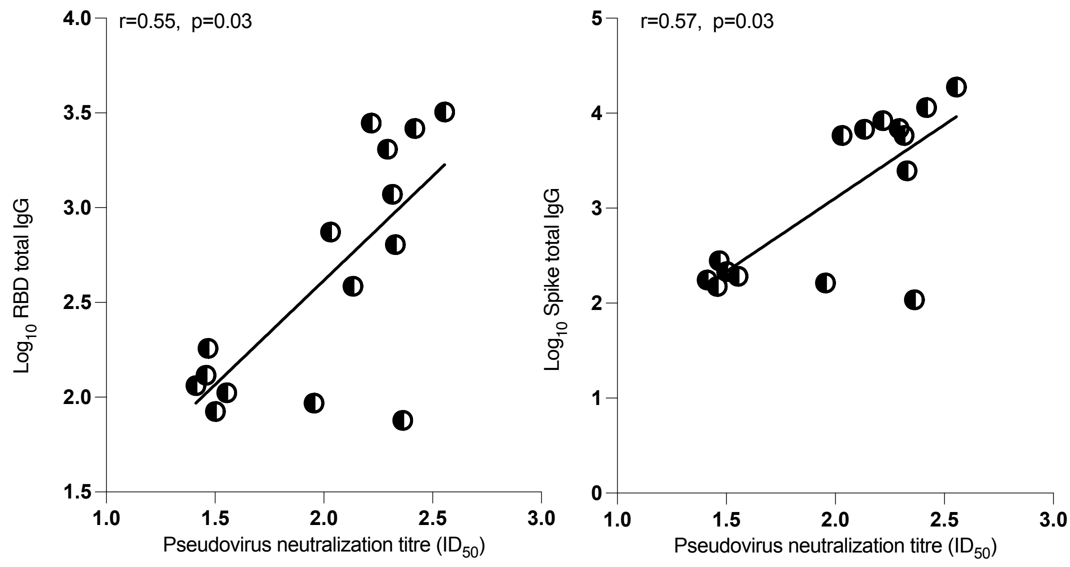

Supplementary Figure 1a: Spearman correlation between binding and neutralizing antibodies prior to vaccination in Nigerian participants. Correlation between receptor binding domain (RBD) IgG binding antibody responses and neutralization by sera against SARS-CoV-2 in a spike lentiviral pseudotyping assay expressing wild-type spike (D614G) **ns**=not significant. These data include only 12 individuals with ID50>20 at baseline and who were Anti-N antibody negative**.** ID50 is expressed as $\log_{10}$. **b. Spearman correlation between binding and neutralizing antibodies prior to vaccination in Ghanaian participants.** Correlation between receptor binding domain (RBD) IgG binding antibody responses and neutralization by sera against SARS-CoV-2 in a spike lentiviral pseudotyping assay expressing wild-type spike (D614G) **ns**=not significant. These data include only 15 individuals with ID50>20 at baseline and who were Anti-N antibody negative**.** ID50 is expressed as $\log_{10}$

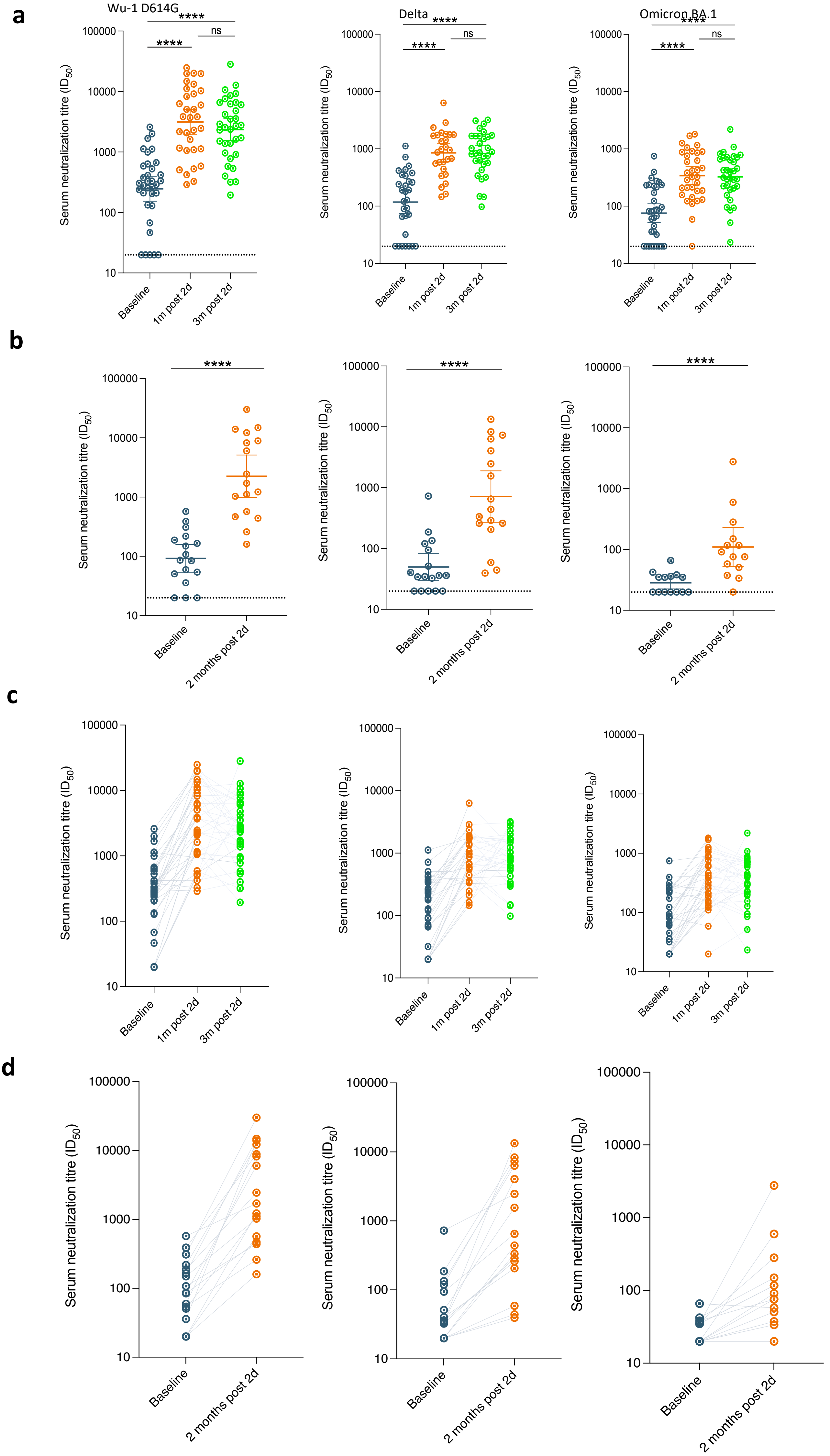

Supplementary Figure 2: SARS-CoV-2 neutralization by sera from AZD1222 vaccinated individuals who were positive for N antibodies during follow up. Serum neutralization of pseudovirus after two doses of vaccine against pseudotyped virus (PV) expressing wild-type spike protein (D614G), delta and omicron variants of concerns from (n=34) participants at baseline (prior to first dose vaccination), 1m (1 month) after second dose vaccination and 3m (3 months) after vaccination and had ≥1 timepoint where anti-N IgG was positive on study from the Nigerian cohort (**Supplementary Figure 2a and 2c**) and from the Ghanaian cohort (n=17) [(**Supplementary Figure 2b and 2d**). Data are representative of two independent experiments comprising of two technical replicates. . Data points were compared using Wilcoxon test and shown as geometric mean titre (GMT) with 95% confidence interval. *P<0.05; ****P<0.0001; ns=not significant.

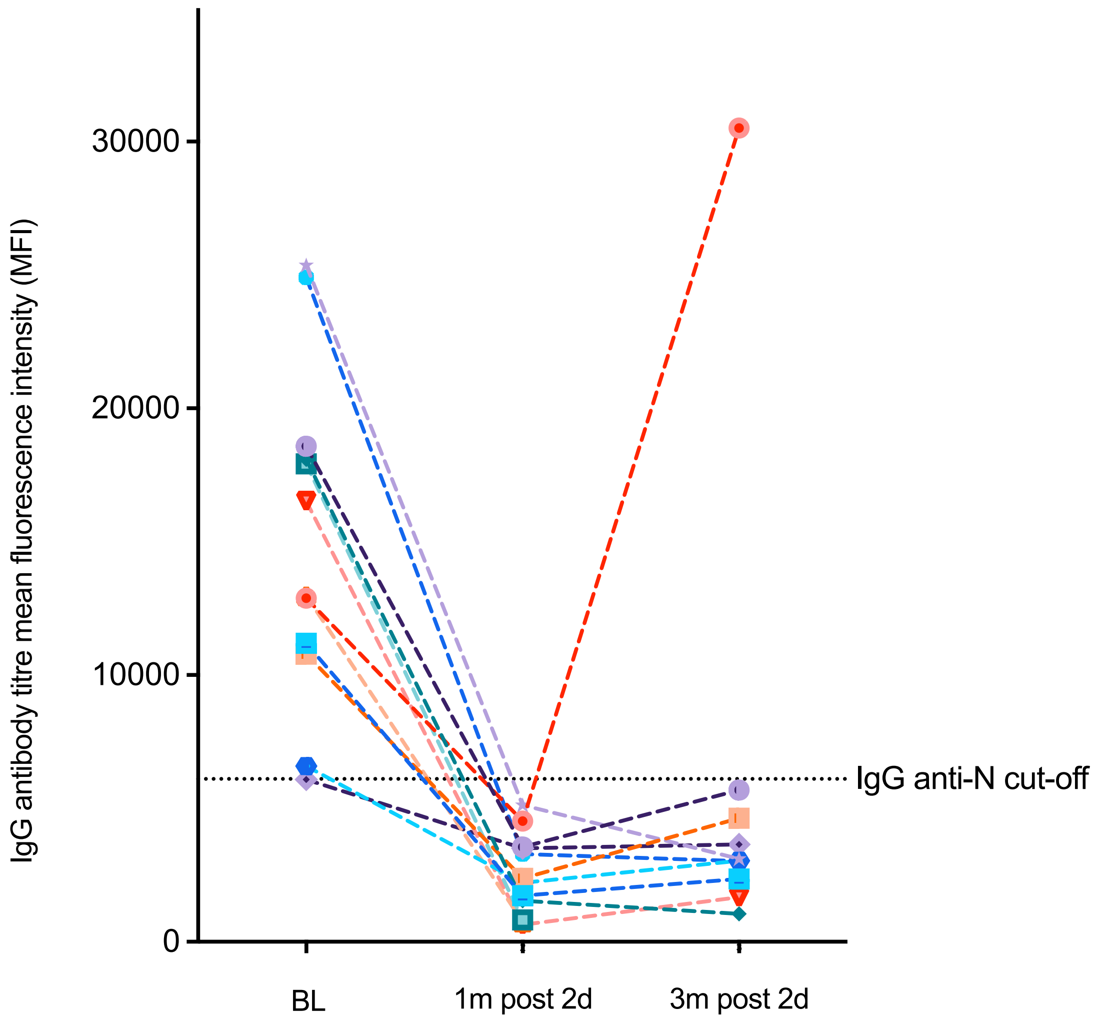

Supplementary Figure 3: Kinetics of SARS-COV-2 total IgG anti-N antibodies in individuals positive for N at baseline from the Nigerian cohort. Note in orange one participant with SARS-COV-2 reinfection following evidence of positive IgG anti-N at baseline; negative IgG anti-N at 1 month after second dose and positive IgG anti-N 3 months post-second dose.

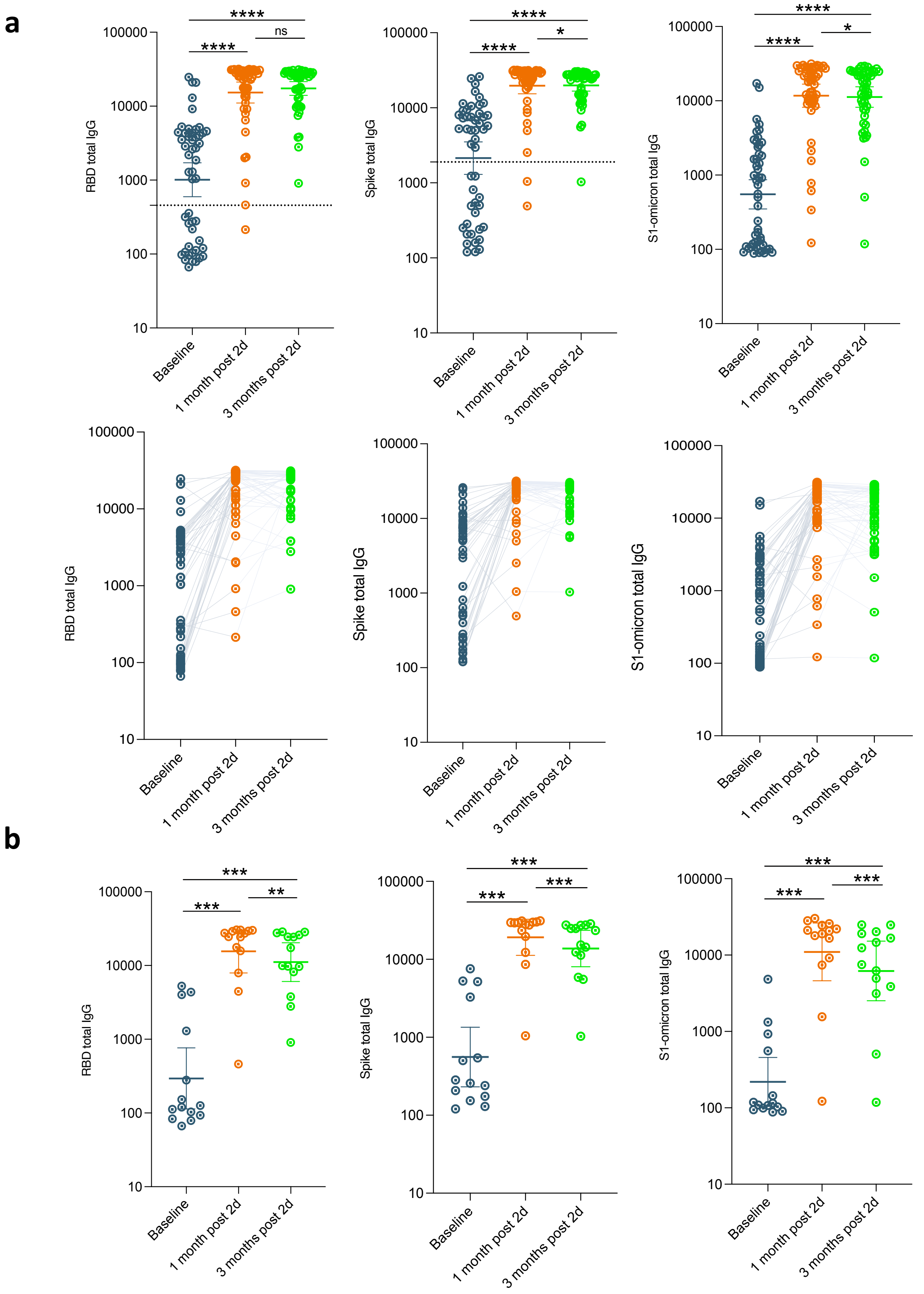

Supplementary Figure 4: SARS-CoV-2 plasma binding antibodies against Wu-1 RBD and Spike S1 and Omicron BA.1 S1 from AZD1222 vaccinated individuals with 3 months follow up. a) Participants at baseline (prior to first dose vaccination), 1m (1 month) after second dose vaccination and 3m (3 months) after vaccination and had ≥1 timepoint where anti-N IgG was positive on study (N=39). **b)** Plasma binding antibodies (n=15) participants at baseline (prior to 1st dose vaccination), 1m (1 month) after 2nd dose vaccination and 3m (3 months) after vaccination in participants who were anti-N IgG negative throughout study period. Data are representative of two independent experiments comprising of two technical replicates. Data shown as mean fluorescence intensity (MFI) with 95% confidence interval. . Data points were compared using Wilcoxon test. *p<0.05; **p<0.01, ***P<0.001, ****p<0.0001; ns=not significant.

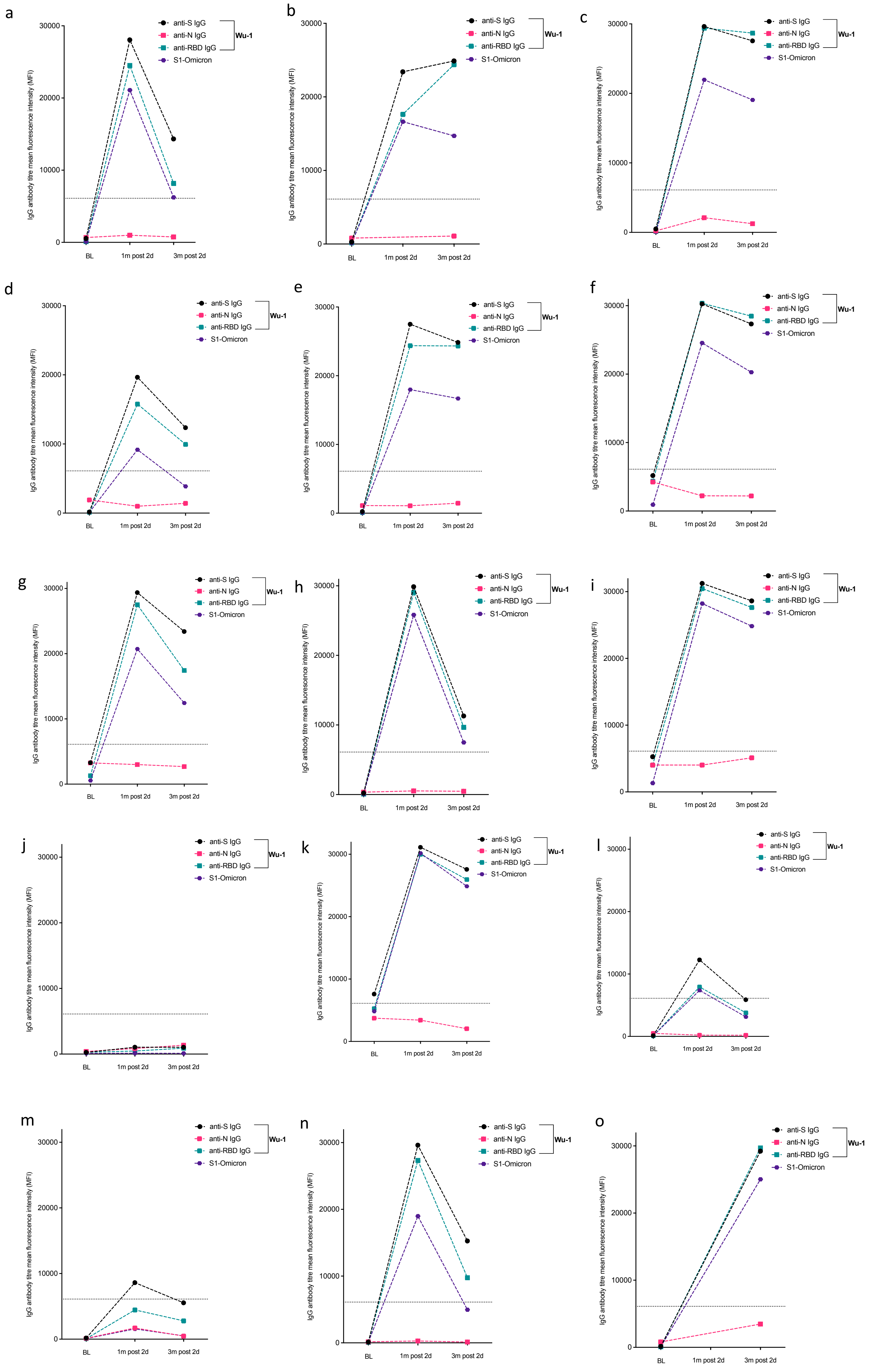

Supplementary Figure 5: Kinetics of anti-SARS-COV-2 IgG binding antibodies to Wu-1 and Omicron in eight participants with no evidence of infection throughout study duration from the Nigerian cohort (a-o), at baseline, 1 month after second dose and 3 months post-second dose. Binding antibodies to Wu-1 and Omicron BA.1 are shown. One subject (o) did not have sufficient sample volume available for testing at 1 month after second dose.

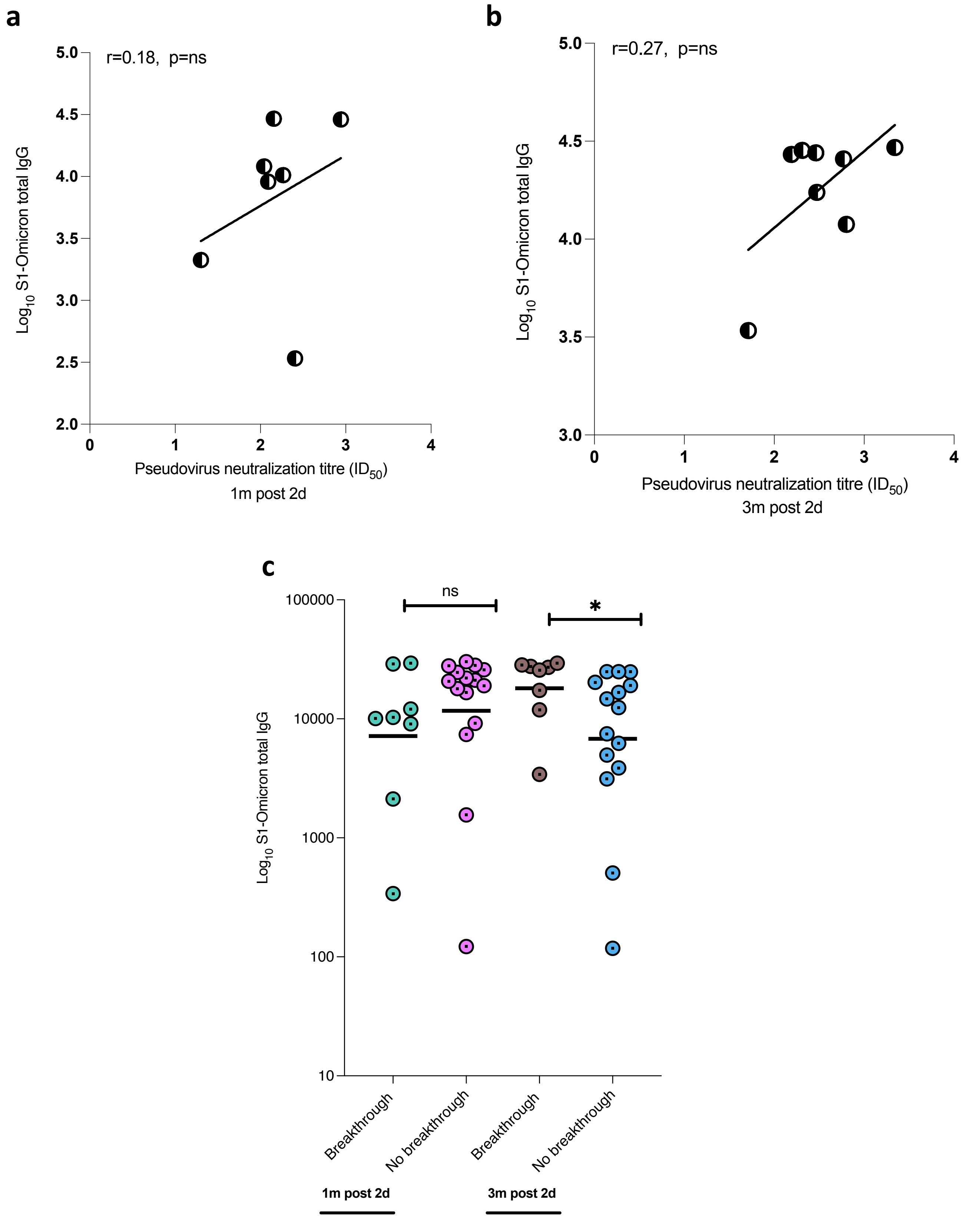

Supplementary Figure 6 and 6b: Spearman correlation between S1-Omicron specific binding and neutralizing antibodies prior in Nigerian participants with breakthrough infection 1 and 3m after second-dose vaccination. Correlation between anti-spike S1-Omicron specific binding IgG binding antibody responses and neutralization by sera against SARS-CoV-2 in a spike lentiviral pseudotyping assay expressing wild-type spike (D614G) **ns**=not significant. These data include only 8 individuals with breakthrough infection**.** ID50 is expressed as $log_{10.}$ **6c:** S1 Omicron specific anti-spike binding IgG levels in individuals with breakthrough infection between 1 and 3 months post vaccination (n=8) and with no evidence of 'natural' infection (n=15). Data points were compared using Wilcoxon test and shown as geometric mean titre (GMT) with 95% CI. Data are representative of two independent experiments comprising of two technical replicates. *P<0.05.
