# Supplementary material for: SARS-COV-2 antibody responses to AZD1222 vaccination in West Africa
Source: Nat Commun. 2022 Oct 17;13:6131. doi: 10.1038/s41467-022-33792-x (PMC9574797; doi:10.1038/s41467-022-33792-x)
Supplement: Supplementary file 3 — Reporting Summary [file 41467_2022_33792_MOESM3_ESM.pdf]

nature portfolio

Corresponding author(s): Ravindra K Gupta

Last updated by author(s): Sep 5, 2022

Reporting Summary

Nature Portfolio wishes to improve the reproducibility of the work that we publish. This form provides structure for consistency and transparency in reporting. For further information on Nature Portfolio policies, see our Editorial Policies and the Editorial Policy Checklist.

Statistics

For all statistical analyses, confirm that the following items are present in the figure legend, table legend, main text, or Methods section.

n/a	Confirmed	
☐	☒	The exact sample size (n) for each experimental group/condition, given as a discrete number and unit of measurement
☐	☒	A statement on whether measurements were taken from distinct samples or whether the same sample was measured repeatedly
☐	☒	The statistical test(s) used AND whether they are one- or two-sided<br>*Only common tests should be described solely by name; describe more complex techniques in the Methods section.*
☐	☒	A description of all covariates tested
☐	☒	A description of any assumptions or corrections, such as tests of normality and adjustment for multiple comparisons
☐	☒	A full description of the statistical parameters including central tendency (e.g. means) or other basic estimates (e.g. regression coefficient) AND variation (e.g. standard deviation) or associated estimates of uncertainty (e.g. confidence intervals)
☐	☒	For null hypothesis testing, the test statistic (e.g. F, t, r) with confidence intervals, effect sizes, degrees of freedom and P value noted<br>*Give P values as exact values whenever suitable.*
☒	☐	For Bayesian analysis, information on the choice of priors and Markov chain Monte Carlo settings
☒	☐	For hierarchical and complex designs, identification of the appropriate level for tests and full reporting of outcomes
☒	☐	Estimates of effect sizes (e.g. Cohen's d, Pearson's r), indicating how they were calculated

Our web collection on statistics for biologists contains articles on many of the points above.

Software and code

Policy information about availability of computer code

Data collection	Graphpad Prism 9.3.1.was used to produce figures
Data analysis	Characteristics of participants were expressed as proportions and percentages for categorical variables and median inter quartile range (IQR) for continuous variables. Mann-Whitney or Wilcoxon test was used to compare neutralization antibody titres across timepoints and compare participants based on IgG anti-N strata.

For manuscripts utilizing custom algorithms or software that are central to the research but not yet described in published literature, software must be made available to editors and reviewers. We strongly encourage code deposition in a community repository (e.g. GitHub). See the Nature Portfolio guidelines for submitting code & software for further information.

Data

Policy information about availability of data

All manuscripts must include a data availability statement. This statement should provide the following information, where applicable:

- Accession codes, unique identifiers, or web links for publicly available datasets
- A description of any restrictions on data availability
- For clinical datasets or third party data, please ensure that the statement adheres to our policy

All data generated or analysed in this study are included in this published article and its Supplementary Information file. Source Data are provided with this article. Data are available without restriction from Prof Ravindra Gupta (rkg20@cam.ac.uk)

Human research participants

Policy information about studies involving human research participants and Sex and Gender in Research.

Reporting on sex and gender	Findings apply gender (Male/Female) as reported by study participants. Consent was provided by patients to provide individual level data.
Population characteristics	We recruited a total of 667 participants from Nigeria and Ghana, of which, 369 were male with median age of 36. (49 (Nigeria) + 45 (Ghana) provided longitudinal sampling were included in the extended analyses. We describe the population characteristics of the patients from which sera was utilized in the extended analyses. This is detailed in Table 1.
Recruitment	Participants who were earmarked to receive at least one dose of the AZD1222 vaccine and volunteered to participate, were recruited into the study in Lagos (Nigerian Institute of Medical Research (NIMR) and Federal Medical Centre, Ebute Metta,) and Kumasi, Ghana (i) Kumasi Centre for Collaborative Research in Tropical Medicine (KCCR) ii) Clinical hostel of the School of Medicine and Dentistry, Kwame Nkrumah University of Science and Technology (KNUST) iv) Kwadaso Seventh-day Adventist Hospital, Kumasi and v) Kumasi South Hospital, Kumasi). All participants available to be vaccinated were recruited and there was no selection bias.
Ethics oversight	This study was approved by the Institutional Review Board of NIMR (IRB-21-040) and the Committee of Human Research, Publication and Ethics of KNUST (CHRPE/AP/091/21)

Note that full information on the approval of the study protocol must also be provided in the manuscript.

Field-specific reporting

Please select the one below that is the best fit for your research. If you are not sure, read the appropriate sections before making your selection.

☒ Life sciences ☐ Behavioural & social sciences ☐ Ecological, evolutionary & environmental sciences

For a reference copy of the document with all sections, see nature.com/documents/nr-reporting-summary-flat.pdf

Life sciences study design

All studies must disclose on these points even when the disclosure is negative.

Sample size	All patients presenting to clinic were recruited; experiments were performed in participants with longitudinal sampling over recruitment period
Data exclusions	No exclusions ; All participants recruited into study were included in the study. Extended analyses was performed only in subjects with available longitudinal sampling.
Replication	Experiments were done in technical duplicates and an independent experimental repeat was successfully performed
Randomization	Not applicable as this was not an intervention study.
Blinding	Not applicable as this was not an intervention study.

Reporting for specific materials, systems and methods

We require information from authors about some types of materials, experimental systems and methods used in many studies. Here, indicate whether each material, system or method listed is relevant to your study. If you are not sure if a list item applies to your research, read the appropriate section before selecting a response.

Materials & experimental systems

n/a	Involved in the study	
☒	☐	Antibodies
☐	☒	Eukaryotic cell lines
☒	☐	Palaeontology and archaeology
☒	☐	Animals and other organisms
☒	☐	Clinical data
☒	☐	Dual use research of concern

Methods

n/a	Involved in the study	
☒	☐	ChIP-seq
☒	☐	Flow cytometry
☒	☐	MRI-based neuroimaging

Eukaryotic cell lines

Policy information about cell lines and Sex and Gender in Research

Cell line source(s)	HEK 293T and Hela-ACE2 cells were used. Sources: 293Ts ATCC: CRL-3216 and HELA-ACE2: Were a kind gift from Dr. James Voss, SCRIPPS
Authentication	None of the cell lines used were authenticated.
Mycoplasma contamination	All cell lines used were tested (by PCR) and were mycoplasma free.
Commonly misidentified lines (See ICLAC register)	No commonly misidentified lines were used in this study.
